# Blunt Laryngeal Fracture Status Post Fall on a Paintball Gun

**DOI:** 10.7759/cureus.2637

**Published:** 2018-05-16

**Authors:** Sanjiv Gray, Khuram Khan, Beatrice Dieudonne, Farhana Iqbal, Saqib Saeed

**Affiliations:** 1 Surgery, University of Central Florida, Orlando, USA; 2 Surgery, Harlem Hospital Center, New York, USA; 3 Internal Medicine, Richmond University Medical Center, Staten Island, USA

**Keywords:** laryngeal fracture, blunt trauma, neck hematoma, thyroid cartilage, flexible laryngoscopy

## Abstract

Laryngeal fracture is a rare but potentially lethal injury. A high degree of suspicion along with an expeditious investigation is warranted to prevent airway complications. The initial assessment of the airway is vital as the patient can suffer a severe anoxic brain injury. If the patient is stable, a computed tomography (CT) scan of the neck should be obtained; this was done urgently for our patient and was managed without any extraneous intervention. We report a case of a 38-year-old man that presented to the emergency room with anterior neck pain after falling onto the back of a paintball gun. The patient was managed conservatively and favorable outcomes were obtained.

## Introduction

Blunt laryngeal fracture after a fall on paintball gun can be fatal if left untreated. Prompt recognition and diagnosis are essential to managing larynx injuries. If treatment is delayed, patients can suffer from voice box injury, swallowing, and other airway patency problems [1]. In emergent situations, providers should understand such entities to manage laryngeal injuries in trauma settings correctly.

## Case presentation

**Figure 1:**
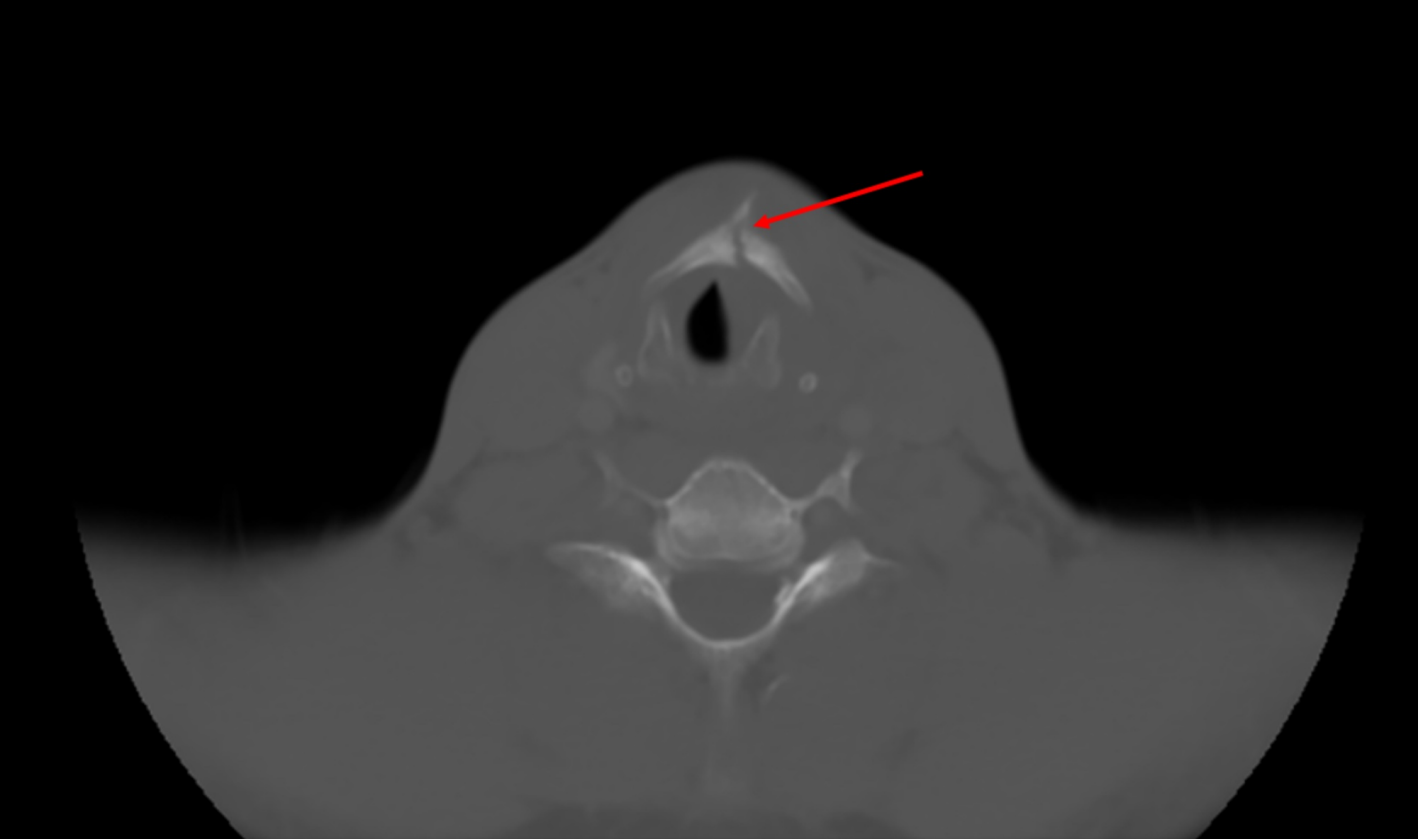
Thyroid cartilage fracture with a pharyngeal hematoma (red arrow)

A 38-year-old man presented to the emergency room with complaints of anterior neck pain. He was playing with a paintball gun when he tripped and fell, landing on the back of the paintball gun and impacting his anterior neck, leaving him with difficulty in breathing, swallowing, and with severe neck pain. On arrival to the emergency room, his pain had improved, and he had no difficulty breathing. When he spoke, his voice was hoarse with some irritation evident. He felt a globus sensation each time he swallowed. On physical examination, a small bruising on the anterior neck at the thyroid cartilage was noted. Additional observations included mild tenderness to palpation, a full range of motion of his neck with no crepitus, no bleeding, no significant swelling of his neck and no palpable cervical lymphadenopathy. Computed tomography (CT) scan of his neck showed a thyroid cartilage fracture with a pharyngeal hematoma on the hypopharyngeal wall on the left effacing the piriform sinus (Figure 1). Upon re-examination, he was in no distress; however, he was admitted to the surgical intensive care unit for close monitoring. Otolaryngology service was consulted, and a flexible nasal laryngoscopy was performed via left nasal cavity. The procedure included advancing a scope down into the nasopharynx; hyperemia of the vocal cords was observed, both vocal cords were mobile, though the left was slightly sluggish. In addition to this, a hematoma on the posterior portion of the left arytenoid into blunting of the left piriform sinus was noted; the rest of the exam was within normal limits. After the procedure, the patient was diagnosed with a closed fracture of the thyroid cartilage with a hematoma to the left piriform sinus and aryepiglottic fold without compromise to the airway. The patient continued to be observed in the surgical intensive care unit and was started on a full liquid diet day one; he advanced as tolerated and was discharged home on hospital day two without any airway issues. He came to out-patient follow-up and reported doing well.

## Discussion

Laryngeal fracture is a rare injury with an incidence of 1 in 30,000 patients [2]. Its rarity is attributed to the sturdy laryngotracheal complex that is relatively shielded in its location between the sternum and the mandible. Due to its infrequent occurrence, increased physician awareness of laryngeal fracture is needed for prompt diagnosis and optimal management. Inappropriate management can have consequences to airway patency, vocalization, and deglutition. The most common causes of laryngeal fracture are blunt trauma secondary to motor vehicle collisions, assault, strangulation, and sports-related trauma. Penetrating trauma was also a cause of laryngeal fracture. Patients usually present with voice changes such as hoarseness, dyspnea, hemoptysis, odynophagia, globus sensation, and dysphagia. Other signs include stridor, anterior cervical edema, ecchymosis, crepitance, loss of thyroid cartilage prominence, and hyoid elevation. CT scan of the neck with intravenous contrast is often used to diagnose the injury and show the extent of the injury while identifying other related pathologies such as a cerebrovascular injury or cervical spine injury. A fiberoptic examination is also used to assess for vocal cord mobility, airway patency, oropharyngeal and endolarynx integrity. Esophageal injury can be investigated using a combination of contrast esophagoscopy and endoscopy. The main tenet of management is to prevent airway compromise and minimize long-term morbidity. Laryngeal mask airway (LMA) would be contraindicated in these cases as the main tenet of management is to secure if the airway is deemed threatened. LMA is a good adjunct but is not considered a secured airway. The LMA sits above the vocal cord and in these cases, one would want a tube distally by either endotracheal intubation or a tracheostomy. LMA may also cause additional trauma to a distorted airway. The optimization of airway management is controversial but difficult; airway algorithms can assist clinicians with decision making, which includes allowing the most skilled physician to attempt the first intervention. A patient who is protecting his airway can be taken to the operating room for laryngoscopic evaluation and fiberoptic intubation as needed versus videoscopic intubation. A qualified surgeon should be available for tracheostomy, if required, during intubation attempt and at laryngoscopy. If the patient is unstable and there is an imminent respiratory arrest, then a cricothyroidotomy will suffice. This can then be converted to a tracheostomy at the time of surgery.

Direct laryngoscopy can be difficult due to distorted anatomy, bleeding, and poor visualization [1]. Some patients will not require any intubation as a part of their non-operative management, but they should be monitored as the development of hematoma or edema may result in airway compromise. Mendelsohn et al. reviewed 564 cases of laryngeal trauma and recommended tracheostomy within 24 hours, which they associated with reduced length of stay in the intensive care unit and the hospital [3]. Restoration of the larynx is recommended to improve long-term voice outcomes. Nondisplaced fractures can be managed nonoperatively but displaced or open fractures should have an operative fixation. Butler et al., in a retrospective review of 112 cases, recommended early diagnosis and optimal management to improve outcomes for airway function, deglutition and functional speech [4]. Early management is defined as within 24 hours. Many protocols call for securing the airway early; each case is unique. Therefore, prompt evaluation and individualized care were recommended [5-6].

Laryngeal fractures are classified by Schafer-Fuhrman Groups who recommended management strategies. These operative techniques are beyond the scope of this review.

Group 1 injuries have minimal mucosal trauma and minor hematomas with no visualized or palpable fracture. Management is nonoperative with observation, humidified air or supplemental oxygen, voice rest and elevation of the head of the bed. Some groups recommend steroids to reduce posttraumatic edema along with antibiotics and antireflux therapy. Group 2 injuries have edema, hematoma, minor mucosal disruption without exposed cartilage, with nondisplaced fracture noted on CT. These require endoscopy to evaluate the full extent of the injury. If there are concerns about progressive edema, endotracheal intubation or tracheostomy should be performed. In cases with normal vocal cord mobility, minor hematoma, minimal mucosal trauma, no exposed cartilage or injury to the anterior commissure, conservative treatment is recommended. Group 3 injuries have massive edema, mucosal tear, exposed cartilage, cord immobility, and/or displaced fracture. Most of these patients will require tracheostomy with repair of the mucosal defects and fixation of the fractures. Group 4 injuries are similar to Group 3 but feature more than two fracture lines or massive trauma to the laryngeal mucosa. Most of these patients will require a tracheostomy with endolaryngeal stent placement. Group 5 injuries have complete laryngotracheal separation and require urgent tracheostomy along with exploration and repair.

## Conclusions

Laryngeal fracture is a rare and potentially life-threatening injury. Early recognition, prompt evaluation, and proper treatment is required for optimal outcomes for functional voice, swallowing, and airway patency. Clinicians should have a low threshold for management and treatment of these injuries.
